# Impaired automatic but intact volitional inhibition in primary tic disorders

**DOI:** 10.1093/brain/awaa024

**Published:** 2020-03-03

**Authors:** Vishal Rawji, Sachin Modi, Anna Latorre, Lorenzo Rocchi, Leanne Hockey, Kailash Bhatia, Eileen Joyce, John C Rothwell, Marjan Jahanshahi

**Affiliations:** Department of Clinical and Movement Neurosciences, UCL Queen Square Institute of Neurology, Queen Square, London, UK

**Keywords:** tics, behavioural inhibition, Tourette, transcranial magnetic stimulation

## Abstract

The defining character of tics is that they can be transiently suppressed by volitional effort of will, and at a behavioural level this has led to the concept that tics result from a failure of inhibition. However, this logic conflates the mechanism responsible for the production of tics with that used in suppressing them. Volitional inhibition of motor output could be increased to prevent the tic from reaching the threshold for expression, although this has been extensively investigated with conflicting results. Alternatively, automatic inhibition could prevent the initial excitation of the striatal tic focus—a hypothesis we have previously introduced. To reconcile these competing hypotheses, we examined different types of motor inhibition in a group of 19 patients with primary tic disorders and 15 healthy volunteers. We probed proactive and reactive inhibition using the conditional stop-signal task, and applied transcranial magnetic stimulation to the motor cortex, to assess movement preparation and execution. We assessed automatic motor inhibition with the masked priming task. We found that volitional movement preparation, execution and inhibition (proactive and reactive) were not impaired in tic disorders. We speculate that these mechanisms are recruited during volitional tic suppression, and that they prevent expression of the tic by inhibiting the nascent excitation released by the tic generator. In contrast, automatic inhibition was abnormal/impaired in patients with tic disorders. In the masked priming task, positive and negative compatibility effects were found for healthy controls, whereas patients with tics exhibited strong positive compatibility effects, but no negative compatibility effect indicative of impaired automatic inhibition. Patients also made more errors on the masked priming task than healthy control subjects and the types of errors were consistent with impaired automatic inhibition. Errors associated with impaired automatic inhibition were positively correlated with tic severity. We conclude that voluntary movement preparation/generation and volitional inhibition are normal in tic disorders, whereas automatic inhibition is impaired—a deficit that correlated with tic severity and thus may constitute a potential mechanism by which tics are generated.

## Teaser

What causes tics? Rawji *et al*. use a combination of behavioural tasks, mathematical modelling and non-invasive brain stimulation to show that voluntary inhibition is unimpaired in patients with primary tic disorders. Conversely, automatic inhibition is impaired, and this impairment correlates with motor tic severity.

**See Jackson and Jackson (doi:10.1093/brain/awaa050) for a scientific commentary on this article.**

## Introduction

The defining character of tics is that they can be transiently suppressed by volitional effort of will, and at a behavioural level this has led to the concept that tics result from a failure of inhibition. However, this logic conflates the mechanism responsible for the production of tics with that used in suppressing them. In fact, the two may differ: tics could be caused initially by increased excitation, whereas they are suppressed at a later stage in their evolution by increasing inhibition. This division is consistent with animal models in which tics are produced by repeated inappropriate activation of striatal medium spiny neurons in the direct pathway. This inhibits the final output neurons of the basal ganglia in the internal segment of the globus pallidus and the substantia nigra pars reticulata, which normally would be tonically active to prevent unwanted movements, thus resulting in disinhibition of the thalamo-cortical targets (Mink, 2001; Israelashvili and Bar-Gad, 2015). Indeed, Israelashvili and Bar-Gad (2015) have proposed that, although the primary deficit lies in the striatum, this only determines the stereotypical spatial expression of the tic, whilst the timing of the tic depends on input arriving from the cortex. In this scenario, volitional suppression of tics could engage inhibitory mechanisms that prevent expression of the nascent movement, by cancelling or compensating for the inappropriate release of pallidal output. Alternatively, they could prevent the initial activation of striatal neurons that produce the output.

Most previous studies of inhibitory control in Tourette syndrome have focused on the former mechanism by measuring performance in tests of reactive and proactive inhibition (Aron, 2011; Jahanshahi *et al.*, 2015). On this basis, patients may detect the premonitory urge that often precedes tics and use reactive inhibition to avoid the tic from manifesting. This principle is applied in habit reversal therapy for tics, which teaches patients to become aware of sensations that precede their tics and to initiate competing movements to the tic (Deckersbach *et al.*, 2014). Conversely, proactive inhibition could increase tonic inhibition of the motor system and increase the threshold for production of tics (Ganos *et al.*, 2018).

Reactive and proactive inhibition have been extensively studied in Tourette syndrome using various tasks such as the Stroop task (Ozonoff and Jensen, 1999), the flanker task, Go/No-Go tasks and the stop-signal task (Ray Li *et al.*, 2006), with mixed results. While some studies report a deficit in inhibitory control (Georgiou *et al.*, 1995; Dursun *et al.*, 2000; Crawford *et al.*, 2005; Ganos *et al.*, 2014), others show no change (Roessner *et al.*, 2008; Jung *et al.*, 2013; Fan *et al.*, 2017) and some an enhanced control (Mueller *et al.*, 2006; Jackson *et al.*, 2007, 2011) relative to age-matched, healthy control subjects (Mazzone *et al.*, 2010; Draper *et al.*, 2014). The lack of agreement suggests that these forms of inhibition are not directly related to production of tics. As outlined in the model above, they may be better understood as mechanisms that can be used to control tic expression.

We have previously argued that tics may result instead from an impairment of ‘automatic’ or ‘habitual’ inhibition that would prevent activation of the striatal focus of tics (Jahanshahi *et al.*, 2015; Jahanshahi and Rothwell, 2017). One version of this view contends that there is a continual input of potential triggers to move from the environment. Such triggers underlie the ‘affordances’ that are engaged when we view an object and automatically select how they are grasped by the hand, or the compulsion to grasp and manipulate objects in patients with anarchic hand syndrome. These potential movements are continually and automatically suppressed by subliminal automatic inhibition, a process that is not subject to voluntary control (Eimer and Schlaghecken, 1998; Sumner *et al.*, 2007; McBride *et al.*, 2018). Without automatic inhibition, motor programmes subconsciously evoked by visual stimuli (Tucker and Ellis, 2004) or internal cues/urges go unchecked. We are not suggesting that all tics are produced by such environmental triggers (although this may be the case in echopraxia), but only that the same automatic process could be used to prevent development of the internal triggers for tics.

The aim of our study was to test this hypothesis by measuring volitional reactive and proactive inhibition as well as automatic inhibition in patients with tics. We used the conditional stop signal task (CSST) to probe volitional reactive and proactive inhibition in a single task. We also tested automatic subliminal inhibition using the masked priming task, which measures how the reaction time to a left/right imperative stimulus is affected by the presentation of an unperceived priming cue. Finally we tested another observation previously made only in children, that patients with Tourette syndrome have reduced corticospinal excitability (CSE) immediately prior to movement onset (Jackson *et al.*, 2013; Draper *et al.*, 2014, 2015). This could also contribute to tic control but could be the result of either automatic or volitional inhibitory mechanisms. The results show that volitional inhibition is intact in patients with tics. Not only do these patients perform normally on the CSST, behavioural modelling also shows that they use the same cognitive strategy as in our control group, while physiological experiments with transcranial magnetic stimulation (TMS) reveal normal evolution of CSE. In contrast, we found clear deficits in automatic inhibition and suggest that these may contribute to the manifestation of tics.

## Materials and methods

### Participants

Nineteen patients with ICD-11 confirmed primary tic disorder [14 male, mean age 35.05 years, standard deviation (SD) 11.96] participated, the majority of whom had Tourette syndrome (*n *=* *16). The first experiment looked at volitional movement execution and inhibition; TMS and performance on the CSST was compared with 15 healthy control participants (13 male, 15 right-handed, mean age 25.53, SD 4.41) who were younger (*t *=* *2.92, *P *=* *0.006, Cohen's *d* = 1.01). In the second experiment looking at automatic motor inhibition, performance on the masked priming task was compared to a different group of 26 healthy control participants (14 male, mean age 29.73, SD 6.35). An unpaired *t*-test showed no significant differences between the age of our patients and these healthy controls (*t *=* *1.93, *P *=* *0.089, *d* = 0.58). Healthy control subjects were recruited from University College London and were screened to ensure that they had no history of physical, neurological or psychiatric illness or drug or alcohol abuse. None were taking any medication that would affect brain function at the time of study.

No participant had contraindications to TMS, assessed by a TMS screening questionnaire. The study was approved by University College London Hospitals Ethics Committee. Informed consent was gained in accordance with the Declaration of Helsinki.

Tic severity was measured with the Yale Global Tic Severity Scale (YGTSS) (Leckman *et al.*, 1989; Storch *et al.*, 2005; Kircanski *et al.*, 2010). Mean motor tic score was 13.05 (SD 4.62) and mean total score was 46.40 (SD 15.40). Eleven patients had a clinical diagnosis of obsessive-compulsive disorder (OCD) and/or attention deficit hyperactivity disorder (ADHD), and six patients were on mood-enhancing or anxiolytic medication, confirmed by examining their medical records. Patient characteristics are presented in Table 1.

**Table 1:** Clinical characteristics of patients with primary tic disorders involved in this study

Patient	Age (years)	YGTSS score					Co-morbidities	Medication
		Motor (/25)	Vocal (/25)	Severity (/50)	Impairment (/50)	Total (/100)		
1	26	24	24	48	0	48	ADHD	Sertraline
2	43	10	8	18	10	28	OCD, ADHD	Clonazepam
3	59	9	0	9	40	49	OCD, anxiety	None
4	38	9	0	9	10	19	OCD, anxiety	Melatonin
5	23	18	18	36	30	66	ADHD, anxiety	Sertraline
6	46	18	13	31	30	61	ADHD, depression	Paroxetine
7	32	5	5	10	30	40	None	Iron
8	30	16	16	32	30	62	None	None
9	44	15	13	28	30	58	OCD, ADHD	None
10	48	9	9	18	20	38	OCD	Citalopram, clonazepam
11	29	8	17	25	20	45	None	None
12	20	17	10	27	30	57	ADHD	None
13	20	12	22	34	40	74	OCD, ADHD, anxiety	None
14	19	15	15	30	20	50	OCD, depression, anxiety	None
15	36	17	15	32	10	42	None	Pimozide
16	28	14	6	20	10	30	None	None
17	26	14	8	22	20	42	None	None
18	49	16	16	32	30	62	None	None
19	50	9	0	9	10	19	None	None

### Behavioural tasks

#### Conditional stop-signal task

**Figure 1:**
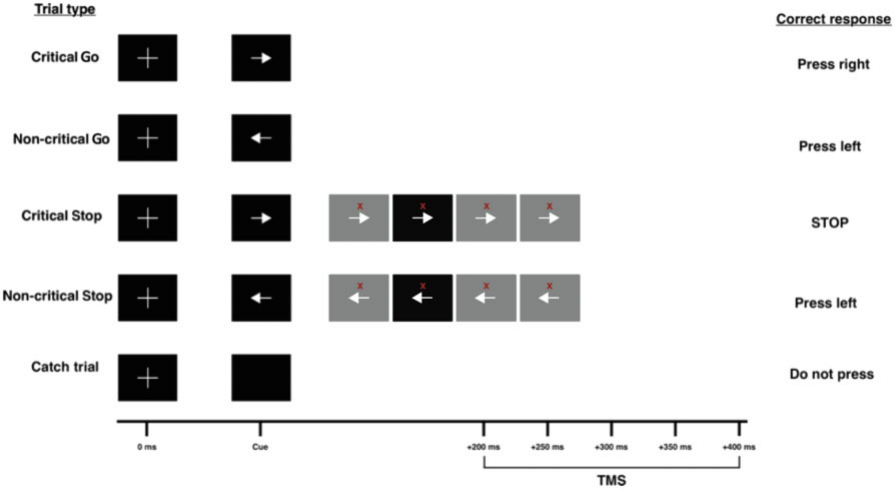
**TMS delivery in the conditional stop-signal task.** Go trials consist of a fixation cross, followed by one of two imperative stimuli (right or left arrow) 500 ms later. In 25% of trials, the Go cue is followed by a Stop signal (red cross) at one of four SSDs (100, 150, 200 or 250 ms after the arrow). Participants are told that one arrow direction is critical and the other is non-critical. Participants must attempt to abort their button press on presentation of a Stop signal after a critical Go cue. If the Stop signal appears after the non-critical Go cue, participants must ignore it and continue pressing the correct button. TMS is delivered on Go trials at one of five time points (counterbalanced and randomized), or 1000 ms into a trial where no signals are shown (baseline trial).

Participants performed two blocks of the CSST (Fig. 1 and Supplementary material), which was driven by custom-made MATLAB (MathWorks) scripts using Psychtoolbox (Brainard, 1997; Pelli, 1997). On each trial, participants were given a warning signal, followed 500 ms later by a left or right pointing arrow, indicating a response with the left or right hand. In different blocks of 120 trials, participants were informed that one of the hands was designated ‘critical’, meaning that on 25% of trials, a Stop signal would appear at different times after the ‘Go’, indicating that participants must refrain from responding. However, if the ‘Go’ signal indicated movement of the other (‘non-critical’) hand, participants were told to ignore the Stop signal and react as usual. Reaction times for movement of the ‘non-critical’ hand are faster than for movements of the ‘critical’ hand because participants tend to delay responding in the ‘critical’ direction, as they expect they will have to stop in some of the trials. This difference in ‘critical’ and ‘non-critical’ Go reaction times is termed the response delay effect (RDE) and serves as a measure of proactive inhibition. The reaction time to the stop signal in ‘critical’ trials (SSRT) is used as a measure of reactive inhibition.

In all trials, a TMS pulse was given to the motor cortex representation for the right first dorsal interosseous muscle at one of five time points (200, 250, 300, 350 and 400 ms) after the Go signal so that we could measure the build-up of CSE prior to movement onset. We could then compare the time course of CSE in ‘critical’ (fast reactions) and ‘non-critical’ (slow reactions) directions. There were also 15 trials, where no signals/cues were presented, serving as catch trials. All trial types were presented in a pseudorandom order. In the 15 baseline trials, TMS was given 1000 ms into the beginning of the trial to assess CSE at rest. Details of TMS and electromyography are given in the Supplementary material.

#### Drift-diffusion modelling

Drift-diffusion modelling (DDM) was applied to reaction times in Go trials to probe why reaction times are longer on ‘critical’ versus ‘non-critical’ trials (using the DMAT toolbox) (Vandekerckhove and Tuerlinckx, 2008). DDM models reactions times as a noisy accumulation of information to a threshold (Ratcliff, 1978; Philiastides *et al.*, 2011). The main parameters of interest are the rate of evidence accumulation (drift rate), the threshold level (boundary separation), and the time taken for stimulus processing and motor execution (non-decision time). For example, reaction times might be longer on ‘critical’ trials because of an increase in boundary separation, or slower drift rate. Further details are given in the Supplementary material.

#### The masked priming task

**Figure 2:**
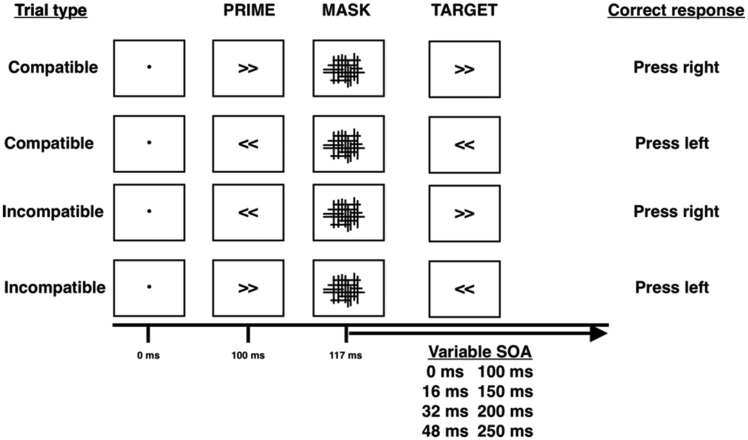
**The masked priming task.** The figure shows the four compatibility trial types in the masked priming task and their appropriate responses. The fixation dot is shown for 100 ms, primes for 17 ms, masks for 100 ms and targets for 100 ms. The onset of the target relative to the mask changes between one of eight interstimulus intervals (0, 16, 32, 48, 100, 150, 200, 250 ms)—stimulus onset asynchrony (SOA).

To assess automatic inhibition, participants performed three blocks of the masked priming task (Fig. 2 and Supplementary material), delivered using the Masked Priming Toolbox (Wilson *et al.*, 2011), using MATLAB (MathWorks) and Psychtoolbox. The task is a modified visual choice-reaction time task, in which participants respond to one of two target stimuli with their right or left hand. In all trials, one of the stimuli (termed a ‘prime’) is presented for 100 ms at different times before target onset. This prime can be either the same (compatible) or different (incompatible) to the target, but is not perceived by participants because it is followed by a masking stimulus. The unperceived prime can either speed (a positive compatibility effect, PCE) or delay (negative compatibility effect, NCE) the reaction time to the target, depending on the time interval between them. The NCE is regarded as a measure of automatic motor inhibition (Eimer and Schlaghecken, 2003).

We were also interested in errors made, categorized as: (i) discrimination (incorrect target selected); (ii) omission (responses >1 s or no button pressed); (iii) fast (response before target presentation); and (iv) premature (responses <150 ms after the target, believed to be responding to the prime).

### Statistical analysis

#### Do proactive and reactive inhibition in patients with tic disorders differ from healthy control subjects?

We predicted that proactive and reactive inhibition would be intact in tic disorders relative to healthy controls. Because of the differences in age between our tic disorders and healthy control groups, we performed an ANCOVA with the covariate Age when comparing the RDE and SSRT between patients and healthy controls. We also performed Spearman’s rank correlation coefficients between YGTSS scores and the SSRT/RDE.

#### Are strategic adaptations for reactive and proactive inhibition similar for patients with tic disorders and healthy control subjects?

We performed three separate (boundary separation, drift rate and non-decision time) two-way repeated measures ANCOVAs with main factors Condition (critical/non-critical) and Group (tic disorders/healthy controls) to assess whether there were any differences between DDM parameters. We used Age and critical and non-critical Go reaction time as covariates due to differences in reaction time and age between groups. Post-hoc *t*-tests were used to evaluate any significant interactions from the ANCOVA and Spearman’s rank correlation coefficients between YGTSS scores and DDM parameters were performed.

#### Do movement preparation and execution differ between patients with tic disorders and healthy control subjects?

Motor-evoked potentials (MEPs) at each time point were collapsed into a grand average. We used a repeated measures ANCOVA with main factors Condition (critical/non-critical), Group (tic disorders/healthy controls), Time from cue (200, 250, 300, 350 and 400 ms) and covariate Age to assess differences in motor preparation between patients with tic disorders and healthy controls (stimulus-locked analysis). *Post hoc* paired *t*-tests were performed between MEPs to probe any significant interactions.

To assess CSE during movement execution between patients with tic disorders and healthy control subjects, we controlled for reaction time differences by calculating the time difference between TMS delivery and reaction time for each trial (response-locked analysis). MEPs were then categorized into 50-ms time bins and a repeated measures ANCOVA was performed with main factors Group (tic disorders/healthy controls), Time before response (200–250, 150–200, 100–150, 50–100 and 0–50 ms) and Condition (critical/non-critical), again adjusting for Age. As per the findings that premovement excitability is lower in children with Tourette syndrome (Jackson *et al.*, 2013; Draper *et al.*, 2014, 2015), we performed *t*-tests between CSE at 0–50 and 50–100 ms time bins, between patients with tic disorders and healthy control subjects. We carried out Spearman’s rank correlation coefficients between YGTSS scores and CSE prior to movement.

#### Is there an impairment of automatic inhibition in patients with tic disorders?

A three-way repeated measures ANOVA with factors Compatibility (compatible/incompatible), stimulus onset asynchrony (SOA) (0, 16, 32, 48, 100, 150, 200, 250 ms) and Group (tic disorders/healthy controls) was used to probe any significant interactions. Paired *t*-tests were used to investigate differences between compatibility within tic disorders and healthy controls. Another ANOVA with variables: Compatibility effect and Group (tic disorders/healthy controls) was carried out to investigate differences in priming effects between patients and healthy control subjects. Compatibility effect was calculated by subtracting the mean reaction time on incompatible trials from the mean reaction time on compatible trials, for each SOA, regardless of the imperative direction. We were particularly interested in compatibility effects for SOAs of 100 ms and 150 ms, where automatic inhibition is believed to operate (Eimer and Schlaghecken, 2003). A one-way ANOVA was then used to probe specific differences between compatibility effects between groups. We used unpaired *t*-tests to assess differences in error rate between groups and calculated Spearman’s rank correlation coefficients between errors made and YGTSS tic scores.

### Data availability

The data supporting these findings are available from the corresponding author.

## Results

### Conditional stop-signal task

#### Does proactive and reactive inhibition in patients with tic disorders differ from healthy control subjects?

Table 2 shows behavioural measurements. Reaction times were ∼90 ms longer for patients with tic disorders than healthy control subjects (Supplementary material). Both patients and healthy controls had faster reaction times on failed Stop trials than on critical Go trials, confirming that they correctly performed the task and that the race model was not violated (Supplementary material) (Verbruggen *et al.*, 2019). Proactive inhibition, as indexed by the RDE, was present and did not differ between tic disorders and control groups (Supplementary material).

**Table 2:** Behavioural measures from the conditional stop-signal task for patients with tic disorders and healthy control subjects

		Primary tic disorder patients		Healthy controls	
Measure	Measure description	Right hand rule		Right hand rule	
		Critical	Non-critical	Critical	Non-critical
Critical Go	RT to Go stimulus in the critical direction	501.64 (77.31)	494.65 (76.48)	410.01 (56.40)	397.10 (53.93)
p(inhibit)	% correct inhibition	62.39 (18.20)	61.32 (19.64)	45.90 (14.97)	46.82 (16.62)
Stop Respond	RT on failure to stop trials	419.00 (77.14)	461.22 (95.44)	375.89 (41.10)	352.38 (46.72)
Go error	% of Go discrimination errors	1.14 (1.94)	1.18 (1.85)	0.67 (1.10)	0.39 (0.88)
Stop signal delay	Delay between Go and Stop signals	190.26 (38.59)	185.00 (41.51)	149.50 (41.49)	150.67 (44.00)
SSRT	Estimated time taken to abort response	334.98 (89.63)	332.30 (87.00)	229.76 (43.12)	223.90 (46.67)
Non-critical Go	RT to Go stimulus in the non-critical direction	429.72 (64.95)	457.60 (103.73)	340.86 (39.37)	355.88 (39.08)
Response delay effect	(Critical Go) − (Non-critical Go) RT	71.93 (58.48)	37.05 (17.67)	69.15 (42.02)	41.22 (33.96)

RT = reaction time. Results are shown as mean (SD).

Patients correctly inhibited on more Stop trials than the control group (Supplementary material). However, this is unlikely to affect the calculation of the SSRT as we used the integration method, which accounts for deviances of p(inhibit) of 50% (Verbruggen *et al.*, 2013). The SSRT was shorter for the healthy control group than patients (Supplementary material) suggesting impaired reactive inhibition in tic disorders. As it is known that patients with OCD or ADHD have impaired reactive inhibition, we hypothesized that this could contribute to the longer SSRTs in patients. When separating patients into those with OCD (*n *=* *7) and those without (*n *=* *12), we found that the group with OCD displayed impaired/delayed reactive inhibition (mean: 367.18 ms, SD: 74.55 ms) compared to healthy control subjects (*t *=* *5.66, *P *<* *0.001, *d* = 2.59), whereas the group without OCD (mean: 316.20 ms, SD: 95.24 ms) did not (Supplementary material). There was no significant effect of OCD status on proactive inhibition compared to healthy controls and there were no differential effects of ADHD, depression and anxiety status or mood-enhancing medication use on reactive or proactive inhibition (Supplementary material). We also found no statistically significant correlations between YGTSS scores and markers of proactive and reactive inhibition (Supplementary material).

### Drift-diffusion modelling

#### Are strategic adaptations during proactive inhibition similar for tic disorders and healthy controls?

**Figure 3:**
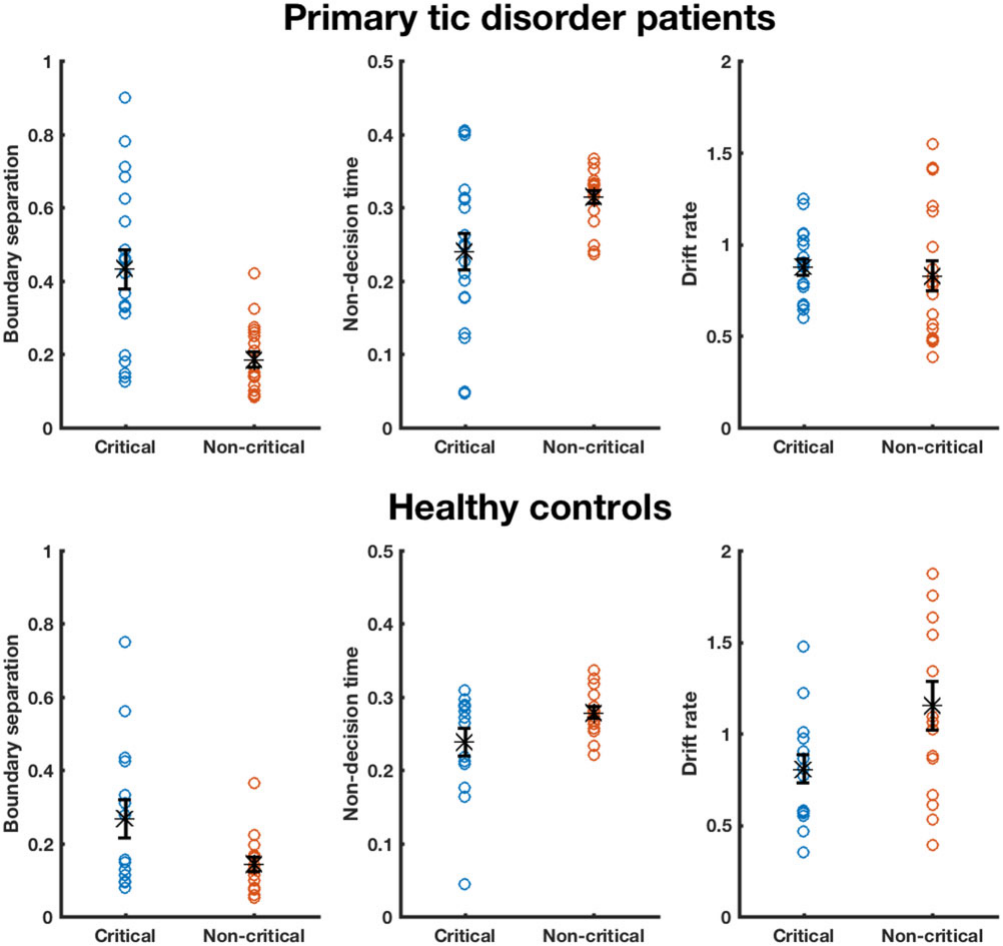
**Drift-diffusion model parameters for Go trials of the conditional stop-signal task.** Estimated DDM parameters are shown for individual participants, for boundary separation, non-decision time and drift rate, for critical and non-critical Go trials performed with the right hand. *Top* panel shows estimated parameter for patients with tic disorders and *bottom* for healthy control subjects. Black stars represent mean parameter estimation, and error bars reflect standard error of the mean (SEM). One healthy control participant’s data were removed because of a drift rate that was >2 SD greater than the mean.

There were significant main effects of Condition for boundary separation [*F*(1,28) = 5.51; *P *=* *0.026, η^2^ = 0.16] and drift rate [*F*(1,28) = 5.15; *P *=* *0.031, η^2^ = 0.16]. These were accompanied by statistically significant effects of Group for boundary separation [*F*(1,28) = 7.14; *P *=* *0.012, η^2^ = 0.20] and non-decision time [*F*(1,28) = 4.24; *P *=* *0.049, η^2^ = 0.13]. The difference between groups is due to the absolute values of boundary separation being greater for patients than controls, probably related to longer reaction times (and non-decision time) in the patient group; the ANCOVA revealed that there was a significant effect of ‘critical go reaction time’ [*F*(1,28) = 4.42; *P *=* *0.045, η^2^ = 0.14] for boundary separation. There were no other statistically significant main effects or interactions (Supplementary material). Both patients with tic disorders (*t *=* *4.39, *P *<* *0.001, *d* = 1.40) and controls (*t *=* *2.75, *P *=* *0.017, *d* = 0.79) increased boundary separation in the face of potential stopping on critical trials relative to non-critical trials (Fig. 3). We found no significant effects of OCD [*F*(1,15) = 0.17; *P *=* *0.687, η^2^ = 0.01] or ADHD [*F*(1,15) = 0.01; *P *=* *0.933, η^2^ = 0.28] status on the DDM parameters during the CSST. No significant correlations between DDM parameters and YGTSS scores were found (Supplementary material).

### TMS evaluation of corticospinal excitability

#### Does movement preparation and execution differ between patients with tic disorders and healthy controls?

##### Evolution of cue-locked corticospinal excitability

**Figure 4:**
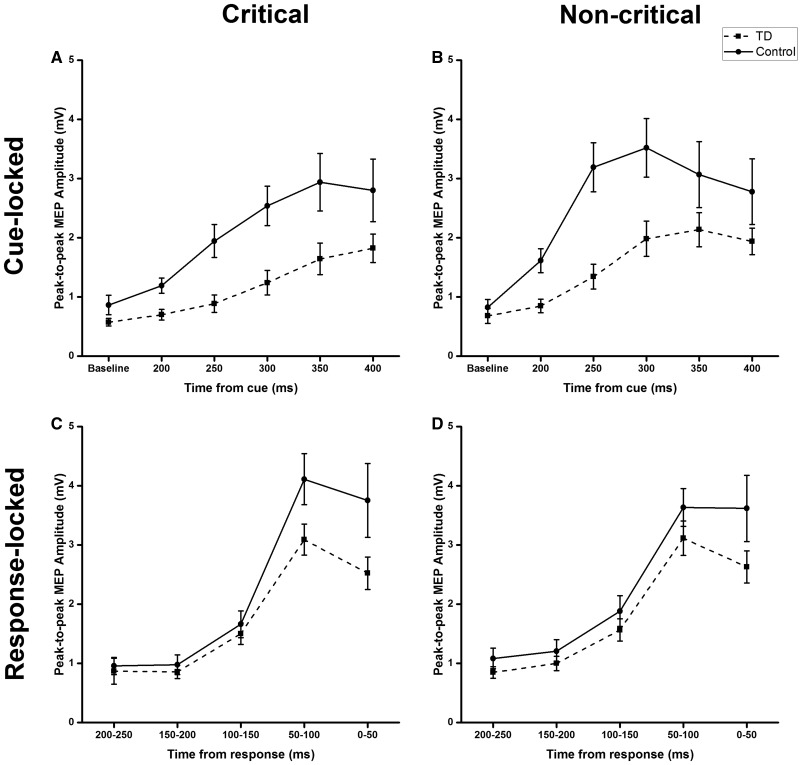
**Stimulus and response-locked MEPs for patients and healthy controls during critical and non-critical Go trials in the conditional stop-signal task.** Cue-locked: MEP amplitudes are plotted against the time at baseline and from stimulus presentation for Go trials in the critical direction (**A**) and non-critical trials (**B**). Response-locked: MEP amplitudes are plotted in 50-ms time bins determined by the time between TMS and response, such that smaller values represent data points closer to responses. Plots on each graph represent CSE from patients and controls. These are plotted for critical (**C**) and non-critical (**D**) Go trials, for patients and healthy control subjects. Error bars represent mean ± SEM. TD = patients with primary tic disorders.

Baseline MEPs did not differ between patients and controls (Supplementary material). There was a slower build-up of CSE after the Go-signal in patients, confirmed by *post hoc t*-tests showing that MEP amplitude was greater for non-critical than critical Go trials at 200, 250, 300 and 350 ms but not 400 ms (Fig. 4A and B, Supplementary material). We found significant main effects of Condition [*F*(1,31) = 7.54; *P *=* *0.01, η^2^ = 0.20] and Group [*F*(1,31) = 5.57; *P *=* *0.025, η^2^ = 0.15] only, with a trend for Time from cue [*F*(4,124) = 2.10; *P *=* *0.085, η^2^ = 0.06]. We found no other significant main effects or interactions (Supplementary material).

##### Evolution of response-locked corticospinal excitability

It is difficult to interpret the results of the cue-locked analysis because of the differences in reaction times between groups. Because of this we aligned the data to response onset (Fig. 4C and D). When the influence of reaction time is removed, there is in fact no difference between groups in the rate of rise of CSE prior to movement in critical and non-critical trials. Thus, there were no statistically significant effects of condition, group or interaction factors (Supplementary material). There was a significant effect of time before response [*F*(4,72) = 9.00; *P *<* *0.001, η^2^ = 0.33]. Even though there was a tendency for CSE to be smaller just prior to movement onset in the tic disorders group, as in Draper *et al.* (2014), this was not statistically significant (Supplementary material), nor was there a correlation between YGTSS scores and excitability prior to movement.

These results indicate that prior to movement execution, preparatory changes in CSE are the same in the control group and patients with tic disorders, suggesting that patients with tic disorders do not have an abnormally excitable motor output.

### Masked priming task

#### Is there an impairment of automatic inhibition in patients with tic disorders?

##### Priming effects in healthy control subjects

**Figure 5:**
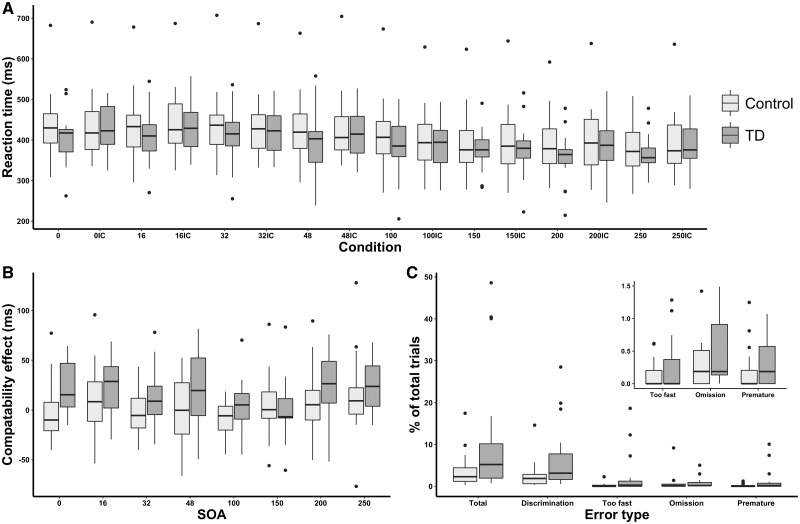
**Priming effects and errors from the masked priming task.** (**A**) Reaction times are plotted for each condition with numbers denoting the SOA (time difference between the mask and target) and letter denoting the compatibility of the prime-target set (C = compatible; IC = incompatible). (**B**) The compatibility effects are shown for each SOA, with values >0 meaning positive compatibility effects and those below 0 meaning negative compatibility effects. (**C**) Box plot showing the errors made on the masked priming task as a proportion of the total number of trials. *Inset:* The differences between groups for too fast, omission and premature errors. TD = patients with primary tic disorders.

A summary of findings from the masked priming task is shown in Fig. 5. We found statistically significant effects of SOA and compatibility but not of group, showing that reaction times between conditions were similar (Supplementary material). As expected from previous work, the control group responded more slowly (NCE) when the prime preceded the target by short intervals (100 ms) whereas their responses were speeded (PCE) if the interval was longer (250 ms). Thus, there was a significant effect of SOA [*F*(7,175) = 80.52; *P *<* *0.001, η^2^ = 0.76] and a SOA × Compatibility interaction [*F*(7,175) = 3.02; *P *=* *0.005, η^2^ = 0.11] but no main effect of compatibility (Supplementary material). *Post hoc* paired *t*-tests showed a significant positive priming effect at an SOA of 250 ms (*t *=* *2.08, *P *=* *0.048, *d* = 0.22) and the NCE (marker of automatic motor inhibition) at 100 ms (*t *=* *2.46, *P *=* *0.021, *d* = 0.10).

##### A positive compatibility effect, but no negative compatibility effect is present in patients with tic disorders

In contrast to the control group, the prime never slowed responses (NCE) in the patients with tic disorders at any SOA. There were significant effects of SOA [*F*(7,119) = 52.28; *P *<* *0.001, η^2^ = 0.76], Compatibility [*F*(1,17) = 18.06; *P *=* *0.001, η^2^ = 0.52] and SOA × Compatibility [*F*(7,119) = 2.72; *P *=* *0.012, η^2^ = 0.14]. We found PCEs at 0, 16, 32, 48, 200 and 250 ms SOAs (Supplementary material). By contrast, the NCE was not observed in our patient sample at 100 ms (*t *=* *0.66, *P *=* *0.515, *d* = 0.06) or 150 ms (*t *=* *0.14, *P *=* *0.892, *d* = 0.02). Lack of the NCE suggests that automatic motor inhibition is impaired in patients with tic disorders. A one-way ANOVA found that PCEs were larger for patients with tic disorders than healthy controls at SOAs of 0, 32 and 48 ms, with statistical trends at 16 and 200 ms (Supplementary material). We also found a statistical trend that the compatibility effect was greater for patients than healthy controls at 100 ms (Supplementary material). We found no differences in priming effects when patients were stratified by OCD/ADHD/depression/anxiety status or mood-enhancing medication use (Supplementary material).

##### Patients with tic disorders make more errors than healthy control subjects, consistent with impaired automatic inhibition

Patients made more total (*t *=* *2.51, *P *=* *0.016, *d* = 0.77), discrimination (*t *=* *2.43, *P *=* *0.019, *d* = 0.76), fast (*t *=* *2.52, *P *=* *0.032, *d* = 0.67) and premature (*t *=* *2.14, *P *=* *0.038, *d* = 0.64) errors than healthy controls. As fast and premature errors both reflect responding impulsivity, we combined these errors, which were more prevalent in patients than controls (*t *=* *2.20, *P *=* *0.034, *d* = 0.65). Patients and controls did not differ in the number of omission errors (*t *=* *0.48, *P *=* *0.634, *d* = 0.14).

If patients fail to inhibit responses to the prime, then they should make more discrimination errors during incompatible than compatible prime-target combinations. If not true, then discrimination errors should be equally distributed between incompatible and compatible trials. We found that more discrimination errors were made on incompatible than compatible trials by patients (*t *=* *2.75, *P *=* *0.014, *d* = 0.55) but not controls (*t *=* *1.40, *P *=* *0.173, *d* = 0.29). One possibility is that patients prioritized speed over accuracy, despite being told to aim for both. However, as indicated by our initial analysis, there was no significant effect of group on reaction times during the masked priming task (Supplementary material). We found no differences in error rates when patients were stratified by OCD/ADHD/depression/anxiety status or mood-enhancing medication use (Supplementary material).

These results suggest that patients with tic disorders exhibit an impairment to inhibit responses to the prime in the masked priming task—a manifestation of an impairment in automatic motor inhibition.

##### Errors consistent with an impairment of automatic inhibition are positively correlated with tic severity

We investigated whether errors correlated with the clinical severity of tics by calculating Spearman’s rank correlation coefficients between each of the errors made and YGTSS tic severity scores. Tic severity correlated with total (r_s_ = 0.50, *P *=* *0.036), discrimination (r_s_ = 0.52, *P *=* *0.026), fast (r_s_ = 0.48, *P *=* *0.046), premature (r_s_ = 0.56, *P *=* *0.017) and total fast ‘impulsive’ errors (r_s_ = 0.57, *P *=* *0.014) but not with omission errors (r_s_ = 0.11, *P *=* *0.673). We found similar results when correlating with motor and vocal scores, and reassuringly, found no correlations with tic impairment—a subjective measure of how tics affect daily life (Supplementary material).

## Discussion

As predicted (Jahanshahi *et al.*, 2015; Jahanshahi and Rothwell, 2017), we found that volitional inhibition, as measured behaviourally by proactive and reactive inhibition in the CSST, was normal in patients with tic disorders, relative to healthy control subjects, whereas automatic inhibition on the masked priming task was impaired. DDM of the CSST confirmed that the strategy used to produce proactive inhibition was the same in patients and controls. In addition, the output from motor cortex during movement preparation and execution was the same in patients with tic disorders and control subjects. Finally, no measures of volitional inhibition (SSRT, RDE, CSE or DDM parameter) correlated with tic severity. Together, these results point towards intact volitional inhibition and movement preparation/execution in tic disorders. As noted in the Introduction, although these mechanisms may be used during volitional inhibition of tics, the present results show that they are not directly related to the production of tics. In contrast, in the masked priming task, we found no evidence for an NCE in tic disorders, whereas it was present in the control group. Furthermore, patients made more errors than control subjects, and these errors were more consistent with an inability to inhibit the prime—a feature of impaired automatic inhibition. Interestingly, the error rate in patients was significantly correlated with tic severity. We conclude that patients with tic disorders have impaired automatic inhibition.

### Proactive and reactive inhibition are intact in patients with tic disorders

Initial analysis showed that SSRT was longer/delayed in patients with tic disorders than in healthy control subjects, which implies a problem in reactive inhibition. However, this effect was driven by delayed reactive inhibition in the seven patients with tic disorders who had OCD—something found in studies of response inhibition in OCD (Chamberlain *et al.*, 2005, 2007; Menzies *et al.*, 2007). The tic disorders sample without OCD did not have longer/delayed SSRTs relative to the healthy control subjects, and thus had normal reactive inhibition. As mentioned in the 'Introduction' section, reports of motor response inhibition in Tourette syndrome have been conflicting—some report increases, decreases and no change in the SSRT. It may be the case that this heterogeneity comes from not accounting for the co-morbidities that come with Tourette syndrome, namely ADHD and OCD.

The pattern of the RDE was similar to that in healthy controls, suggesting that, like healthy control subjects, patients were able to strategically and adaptively prolong responding in anticipation of an upcoming Stop signal on critical trials. Only one study has previously assessed proactive inhibition in patients with tic disorders (Mancini *et al.*, 2018), finding it normal in children with ‘pure’ (non-OCD) motor tics. Although the previous study was on children with Tourette syndrome, these findings together may suggest that volitional inhibition is retained throughout the disease course.

Although reaction times were slower in our patient sample than in our healthy control group, we believe that this does not change the interpretation of our results. Indeed, we accounted for differences in age and reaction time by statistical adjustments for all analyses in which the CSST was used. Furthermore, our behavioural measures of interest from the CSST (RDE and SSRT) are independent of absolute reaction time values. That is, the RDE and SSRT are both calculated from within a participant’s reaction time distribution; for both, task adaptations account for the absolute reaction time differences within subjects. The RDE is the reaction time difference when stopping might be required and the task design means that the SSD tracks Go reaction time distributions. In fact, it is this same feature of the CSST that makes it suitable in disorders where reaction times are prolonged, such as Parkinson’s disease (Obeso *et al.*, 2011, 2014; Manza *et al.*, 2017).

### Patients and control subjects use the same strategy to mediate proactive inhibition

Interrogation of the strategy used during Go trials with DDM analyses revealed that boundary separation was raised in tic disorders when stopping might be required, a feature seen in the healthy controls too. Overall, it seems that patients use the same strategy to employ proactive inhibition as healthy control subjects. To our knowledge, this is the first report of how strategy is changed when stopping might be required in patients with tic disorders. Although basal ganglia dysfunction is implicated in the pathogenesis of tics, this has been predominantly localized to the striatum (Kalanithi *et al.*, 2005; Bronfeld and Belelovsky, 2011; McNaught and Mink, 2011; Yael and Vinner, 2015). On the other hand, there is accumulating evidence that the subthalamic nucleus mediates the change in boundary separation under restraint (Frank, 2006; Frank *et al.*, 2007; Obeso *et al.*, 2014; Herz *et al.*, 2016). Our data, therefore, support the proposal that subthalamic nucleus function is retained in tic disorders. The absolute values of boundary separation were greater for patients with tic disorders than healthy control subjects. As the drift rates are similar between patients with tic disorders and healthy controls, it is likely that the higher boundary separation in patients is a mathematical consequence of the model to account for their longer reaction times. Indeed, this also predicts that the variation in reaction times for higher boundary separations would be greater—something we observed in our data. As noted above, there was a significant effect of critical Go reaction time on boundary separation, suggesting that the longer reaction times in patients was, in part, mediating this increase in boundary separation.

### Movement preparation and execution are very similar in patients and control subjects

We found that rise in CSE after the cue was slower in patients than controls. However, this was confounded by the fact that patients’ reaction times were slower than the control group. To remove this factor, we carried out a response-locked analysis of CSE, which showed that the rise of excitability prior to movement did not differ significantly between the two groups. This differs from previous data in a Go/No-Go task where a lower CSE was found prior to movement onset in Tourette patients (Heise *et al.*, 2010; Draper *et al.*, 2015). The study by Draper *et al.* (2015) was on a group of adolescents with Tourette syndrome. Tic control generally improves with age (Müller-Vahl, 2009; Scahill *et al.*, 2014; Ganos, 2016) and so their results may not be directly applicable to our adult sample. Indeed, it has been suggested that reduction of CSE is related to the ability of children to control their tics (Draper *et al.*, 2015). It may be the case that successful CSE suppression determines whether children eventually outgrow their tics. Consequently, this may mean that adults with tic disorders are those with less successful CSE suppression.

In summary, behavioural data, modelling and physiology converge on the conclusion that volitional movement preparation, execution and inhibition are normal in patients with tic disorders. This is consistent with the notion that tics are involuntary movements that have a different mechanism than voluntary movements (Obeso *et al.*, 1981; Karp *et al.*, 1996; Bohlhalter *et al.*, 2006).

### Automatic inhibition is impaired in patients with tic disorders

The masked priming task explored both positive and negative priming in patients with tic disorders. Patients exhibited positive priming both at very short and long SOAs that was stronger than we observed in the control group (Aron *et al.*, 2003; Eimer and Schlaghecken, 2003; Seiss and Praamstra, 2006; Sohrabi and West, 2009; D’Ostilio *et al.*, 2012). However, the NCE, a marker of automatic inhibition, was absent in our patient population although it was present in our healthy controls. Our analysis of errors strengthened this hypothesis, showing that patients made more errors than healthy controls: patients made more discrimination, fast and premature errors, all of which point towards patients being unable to inhibit the prime. These effects were not due to patients prioritizing response speed above accuracy as they had similar reaction times to the control group. We conclude that automatic inhibition is impaired in tic disorders.

In contrast to the present results, Stenner *et al.* (2018) reported normal automatic motor inhibition in tic disorders at the single SOA (183 ms between prime and target) they investigated. As the authors themselves noted, they did not investigate the full range of SOA, which is necessary to assay the range of priming effects. In our study not only did we fail to observe a significant NCE in patients with tic disorders, but we also saw that the PCE was larger than controls at both very early and later SOAs, strengthening the case for an impairment of automatic inhibition. Indeed, the positive correlation between errors and tic severity is highly suggestive of a deficit in automatic inhibition, particularly since correlations were specific for automatic inhibition errors whereas omission errors were not correlated with tic severity.

In the model of Eimer and Schlaghecken (2003), the NCE arises from feedforward inhibition in visual facilitation of movement: the prime initially activates neural representations of the target, and if the target appears shortly afterwards, the response is facilitated. However, the prime also activates a parallel inhibitory process that takes longer to activate, that when develops, suppresses activity and impairs response to the target. Effectively it balances out the facilitation and thus reduces noise in the system (Schlaghecken and Eimer, 2002). Reduced excitability of this inhibitory process in patients with tic disorders would result in increased noise in the motor system as posited in the ‘motor noise’ hypothesis (Misirlisoy *et al.*, 2015).

The neural substrate for this impairment in automatic inhibition is currently unknown, although the putative network implicated in masked priming tasks has been shown to involve a cortico-subcortical network, including the medial prefrontal cortex and striatum (Sumner *et al.*, 2007; D’Ostilio *et al.*, 2012), which overlaps with the fronto-subthalamo-striatal-pallidal network proposed to mediate automatic/habitual and goal-directed inhibition (Jahanshahi *et al.*, 2015). Our findings support a role for a deficit in automatic inhibition when this putative network is mapped onto the frontal lobe and striatal deficits in tic disorders (Bloch *et al.*, 2011; Draper *et al.*, 2015); tics generated from striatal dysfunction may not be suppressed by automatic inhibition. Indeed, it has been found that GABA concentrations in the SMA are elevated in patients with tic disorders (Draper *et al.*, 2014). It may be the case that enhanced SMA GABA in tic disorders inhibits the negative phase of the lateralized readiness potential during masked priming, thereby preventing automatic inhibition.

### Limitations

Although we found no significant effects of co-morbidities or medication use, other than OCD status, our sample sizes in these subgroups were not large. Therefore, these results require confirmation in future studies with larger samples and subgroups with or without co-morbidities. Furthermore, co-morbidity status determined in clinical notes may not have been significant at the time of testing and assessment via validated scales should be used in future studies. Nevertheless, we are reassured that investigations of inhibitory control in anxiety (Li *et al.*, 2009; Neo *et al.*, 2011) and depression (Lipszyc and Schachar, 2010; Fortgang *et al.*, 2016; Palmwood *et al.*, 2017) are repeatedly reported as normal, relative to age-matched healthy controls.

Our study did not include any physiological investigation of automatic inhibition. As per the predictions by Jahanshahi *et al.* (2015), we first wanted to assess whether automatic inhibition was impaired in tic disorders. Having confirmed this behaviourally, subsequent experiments will aim to measure physiological parameters, for example using TMS, to investigate the neural substrates of impaired automatic inhibition in tic disorders.

## Conclusions

The results from the CSST suggest that volitional inhibition and movement preparation and execution are normal in patients with tic disorders. Conversely, the masked priming task suggests deficits in automatic inhibition in patients with tic disorders, indexed by the absence of the NCE and increased errors that are consistent with an impairment to inhibit the subliminal prime. Indeed, errors associated with impaired automatic inhibition correlated with clinical measures of tic severity, whereas all measures associated with voluntary movement, did not. We suggest that intact volitional inhibition allows patients to voluntarily suppress their tics and that the cause of their tics is a lack or deficit of automatic inhibition. The results give some insight into the origins of tics and into how habit reversal therapy and deep brain stimulation for tic disorders might operate to control tics.

## Supplementary Material

awaa024_Supplementary_Data

## Abbreviations

ADHD =: attention deficit hyperactivity disorder
CSE =: corticospinal excitability
CSST =: conditional stop-signal task
DDM =: drift-diffusion model
MEP =: motor-evoked potential
NCE =: negative compatibility effect
OCD =: obsessive compulsive disorder
PCE =: positive compatibility effect
RDE =: response delay effect
SOA =: stimulus onset asynchrony
SSRT =: stop-signal reaction time
TMS =: transcranial magnetic stimulation
YGTSS =: Yale global tic severity scale
