# Supplementary material for: Impaired automatic but intact volitional inhibition in primary tic disorders
Source: Brain. 2020 Mar 3;143(3):906–19. doi: 10.1093/brain/awaa024 (PMC7089661; doi:10.1093/brain/awaa024)
Supplement: awaa024_Supplementary_Data [file awaa024_supplementary_data.pdf]

Supplementary material

Conditional Stop-signal task

Each trial began with a white fixation cross on a black background. After 500 ms, this was replaced with a white right or left arrow (go cue), to which the participant responded with their right or left index finger as fast as possible. On 25% of trials, a stop signal (red cross) appeared above the imperative stimulus at a variable delay after the imperative stimulus. This delay, known as the stop signal delay (SSD), was controlled by a dynamic staircase tracking algorithm, whereby the SSD would change depending on the outcome of the previous stop trial. The starting SSD was always set at 150 ms. If the subject successfully prevented their button press on a stop trial, the next stop trial would have an SSD set 50 ms later making inhibition more difficult; whereas if the participant failed to stop, the next stop trial would have an SSD set 50 ms earlier, making inhibition easier. SSDs therefore changed in a staircase manner between four SSDs (100, 150, 200 and 250 ms). This dynamic tracking algorithm in effect individualizes the stopping process as a function of each participant's reaction time and is useful when comparing patients and healthy controls. Participants were instructed to respond as fast as possible to the go signal and not to delay their responses in anticipation of the stop signal. Furthermore, given the conditional nature of the task, participants were told at the beginning of the block that they must follow the stopping rule for one direction (critical, i.e. stop signal after right arrow as go signal) and ignore it for the other direction (non-critical, i.e. stop signal after left arrow as go signal).

The order of trials was pseudorandomised, such that one in every four trials contained a stop signal. Each block consisted of 120 go trials (60 critical, 60 non-critical) and 40 stop trials (20 critical and 20 non-critical). There were also 15 trials, where no signals/cues were presented, serving as catch trials.

Behavioural measures included Critical Go reaction time, Non-critical Go reaction time, Stop Respond reaction time (reaction time on failed stop trials), average SSD, p(inhibit) (proportion of correct stop trials in the CSST) and response delay effect (reaction time difference between critical go and non-critical go trials). The response delay effect (RDE) is the behavioural index of adaptively slowing down in the face of potential stopping, as stopping is required during critical go trials, but no in non-critical go trials. The RDE is a measure of proactive inhibition on the CSST. The stop signal reaction time (SSRT), the behavioural estimate of speed of stopping to the stop signal on critical trials, was calculated using the integration method (Verbruggen *et al.*, 2013). The SSRT is a measure of reactive inhibition on the CSST.

The masked priming task

Each trial begins with a black fixation dot on a white background. After 100 ms, the prime (<< or >>) is presented for 17 ms (one frame at 60 Hz), after which the mask (a rectangular array of randomly orientated line) is presented for 100 ms. After a variable delay determined by increasing frame numbers on a 60 Hz monitor (0,16,32,48,100,150,200,250 ms), the target stimulus is presented (<< or >>), to which the

participant must respond by pressing the ‘A’ or ‘L’ key on the keyboard for left and right responses, respectively. The variable delay between the mask and the target presentation is known as stimulus-onset asynchrony (SOA). As well as the variable delay between the mask and the target, the congruency of the prime and target is also changed; if prime and target are the same stimulus (<</<< or >>/>>) they are deemed as compatible; whereas if the prime and target stimuli are pointing in different directions (<</>> or >>/<<), the trial is deemed incompatible. Each block consisted of 16 different SOA-compatibility combinations, with five repetitions per condition. Participants performed three blocks of the masked priming task.

On the masked priming task, we measured the go reaction time on each trial, indicated by the time between the target presentation and pressing of the button. This was averaged per SOA-compatibility condition. For reaction time analyses, trials with responses <150 ms and >1000 ms were excluded as anticipations and long responses respectively, and only correct trials were included. The reaction time difference between compatibility conditions for each SOA was calculated to give the compatibility effect. This was deemed the positive compatibility effect (PCE) if reaction times were longer on incompatible trials and the negative compatibility effect (NCE) if the reaction times on incompatible trials were shorter. The NCE has been shown to be a manifestation of automatic motoric inhibition (Eimer and Schlaghecken, 2003). The mean reaction time for all correct trials was also calculated. We also calculated the number of errors, which could be of four types: 1) Discrimination errors (the wrong button was pressed in response to the target), 2) omission errors (responses that were greater than one second long or no button was pressed, 3) fast errors (the participant responded before the target had been presented), and 4) premature errors (participant responded <150 ms after the target, believed to be responding to the prime instead). We also calculated the mean reaction time of the discrimination errors and whether they occurred on trials which were compatible (prime and target the same) or incompatible (prime and target different).

Transcranial Magnetic Stimulation and Electromyography Recordings

Throughout the experiment, participants were seated comfortably in a non-reclining chair, with their right and left index fingers rested over the ‘M’ and ‘Z’ keys on the keyboard. Their forearms were supported using a cushion. Electromyographic (EMG) activity was recorded from the right, first dorsal interosseous (FDI) muscle using 19 mm x 38 mm surface electrodes (Ambu WhiteSensor 40713) arranged in a belly tendon montage. The raw signals were amplified, and a bandpass filter was also applied (20 Hz to 2 kHz (Digitimer, Welwyn Garden City, United Kingdom)). Signals were digitised at 5 kHz (CED Power 1401; Cambridge Electronic Design, Cambridge, United Kingdom) and data were stored on a computer for offline analysis (Spike2 version 8.10, Cambridge Electronic Design, United Kingdom).

Single pulse, monophasic TMS was employed using a Magstim 200^2 stimulator (The Magstim Co. Ltd) connected via a figure-of-eight coil with an internal wing diameter of 70 mm. The hotspot was identified as the area on the scalp where the largest and most stable motor evoked potentials (MEPs) could be obtained for the right first dorsal interosseous (FDI) muscle, using a given suprathreshold intensity. The coil was held

approximately perpendicular to the presumed central sulcus and tangentially to the skull, with the coil handle pointing backwards for postero-anterior (PA) stimulation. Stimulation intensity was set to one whereby resting peak-to-peak MEP amplitude was 0.5 mV.

Application of the drift diffusion model

We allowed the drift rate, boundary separation and non-decision time to vary between condition (critical vs non-critical). The starting point was set to half of the boundary separation given that left/right go cues could appear with equal probability. We only used go trials derived from the right hand in this analysis; hence right-hand responses when the right hand was critical in one block, and right-hand responses when the right hand was non-critical in the other block (for each participant, the critical rule was changed between blocks). We did this so that we could make comparisons between the TMS derived measures for the right hand and behavioural performance on the CSST with the same hand. Furthermore, TMS has been known to modulate reaction time, so we wanted a comparison, which controlled for this.

Supplementary table 1: ANCOVA (adjusted for age) for behavioural measures during the CSST between TD patients and healthy controls

Behavioural measurement	Right hand critical				Right hand non-critical			
	dF	F	p	η^2	dF	F	p	η^2
Critical go RT	1,31	6.478	0.016	0.173	1,31	8.562	0.006	0.216
Non-critical go RT	1,31	10.184	0.003	0.247	1,31	4.210	0.049	0.123
Stop Respond RT	1,31	0.395	0.534	0.013	1,31	0.402	0.511	0.032
SSD	1,31	3.831	0.059	0.110	1,31	2.138	0.154	0.065
P(inhibit)	1,31	7.107	0.012	0.187	1,31	5.736	0.023	0.157
RDE	1,31	0.680	0.796	0.002	1,31	0.205	0.654	0.007
SSRT	1,31	8.58	0.006	0.217	1,31	11.183	0.002	0.265

Supplementary table 2: Paired t-tests comparing Stop respond and Critical go reaction times for TD patients and healthy controls

Comparison	Primary tic disorder patients			Healthy controls		
	**t**	**p**	**d**	**t**	**p**	**d**
**Stop response RT vs Critical Go RT**	5.36	< 0.001	1.07	4.74	< 0.001	0.69

Supplementary table 3: Unpaired t-tests SSRT and RDE between TD patients with and without OCD/ADHD/depression/anxiety, and medication use, against healthy controls

Comparison	SSRT			RDE		
	t	p	d	t	p	d
TD+OCD vs HC	5.66	< 0.001	2.59	0.20	0.861	0.09
TD-no-OCD vs HC	1.93	0.10	0.75	0.32	0.756	0.13
TD+ADHD vs HC	-2.53	0.027	2.37	0.90	0.382	-0.13
TD-no-ADHD vs HC	-4.44	0.001	1.22	-1.51	0.149	0.20
TD-Depression vs HC	4.29	0.001	3.23	1.04	0.314	0.78
TD-no-Depression vs HC	3.85	0.001	1.37	-0.40	0.969	-0.01
TD+Anxiety vs HC	2.40	0.027	1.24	-0.80	0.436	-0.41
TD-no-Anxiety vs HC	4.54	< 0.001	1.69	0.53	0.599	0.20
TD-mood-medications vs HC	5.77	0.001	1.52	0.84	0.428	-0.44
TD-no-mood-medications vs HC	-9.87	< 0.001	1.75	-0.665	0.513	0.30

Supplementary table 4 Spearman's rank correlation between YGTSS scores and measures of reactive (SSRT) and proactive (RDE) inhibition

	SSRT		RDE	
**YGTSS feature**	r_s	**p**	r_s	**p**
**Motor**	-0.40	0.097	0.03	0.902
**Vocal**	-0.16	0.536	0.23	0.361
**Severity**	-0.34	0.174	0.16	0.538
**Impairment**	-0.17	0.514	-0.21	0.401
**Total**	-0.28	0.268	0.02	0.948

Supplementary table 5: ANCOVA, adjusted for age, comparing baseline MEP amplitude between TD and healthy controls

Behavioural measurement	Right hand critical				Right hand non-critical			
	dF	F	p	η^2	dF	F	p	η^2
**Baseline CSE**	1,31	2.773	0.106	0.082	1,31	0.843	0.366	0.026

Supplementary table 6: Results of the repeated measures ANCOVA assessing DDM parameters during critical and non-critical go trials during the CSST for TD and healthy controls. Comparisons are adjusted for age, and critical and non-critical go reaction times.

	Boundary separation				Non-decision time				Drift rate			
**Factor**	**dF**	**F**	**p**	**η^2**	**dF**	**F**	**p**	**η^2**	**dF**	**F**	**p**	**η^2**
**Condition**	1,28	5.508	0.026	0.164	1,28	0.355	0.556	0.013	1,28	5.148	0.031	0.155
**Condition*Age**	1,28	1.463	0.237	0.050	1,28	0.208	0.652	0.007	1,28	0.990	0.328	0.034
**Condition*Critical go RT**	1,28	8.567	0.007	0.234	1,28	0.356	0.556	0.013	1,28	1.001	0.326	0.035
**Condition*Non-critical go RT**	1,28	2.145	0.154	0.071	1,28	0.052	0.822	0.002	1,28	0.089	0.768	0.003
**Condition*Group**	1,28	1.502	0.231	0.051	1,28	2.919	0.099	0.094	1,28	0.237	0.630	0.008
**Age**	1,28	< 0.001	0.989	< 0.001	1,28	0.339	0.565	0.012	1,28	< 0.001	0.997	< 0.001
**Critical go RT**	1,28	4.415	0.045	0.136	1,28	0.902	0.350	0.031	1,28	0.079	0.781	0.003
**Non-critical go RT**	1,28	2.720	0.110	0.089	1,28	0.045	0.833	0.002	1,28	0.459	0.503	0.016
**Group**	1,28	7.141	0.012	0.203	1,28	4.241	0.049	0.132	1,28	0.703	0.409	0.024

Supplementary table 7: Spearman's rank correlation between YGTSS scores and critical drift-diffusion parameters

DDM parameter	Boundary separation		Non-decision time		Drift rate	
**YGTSS feature**	r_s	p	r_s	p	r_s	p
**Motor**	-0.33	0.184	< 0.01	0.997	0.11	0.652
**Vocal**	-0.30	0.225	0.17	0.509	-0.11	0.671
**Severity**	-0.38	0.119	0.06	0.800	-0.16	0.527
**Impairment**	0.33	0.182	-0.51	0.031	-0.23	0.358
**Total**	-0.02	0.932	-0.26	0.297	-0.21	0.394

Supplementary table 8: Results of the repeated measures ANCOVA, adjusted for age, assessing the evolution of cue-locked corticospinal excitability

Factor	dF	F	p	η^2
**Condition**	1,31	7.541	0.010	0.196
**Condition*Age**	1,31	2.085	0.159	0.063
**Condition*Group**	1,31	0.002	0.964	< 0.001
**Time from cue**	4,124	2.101	0.085	0.063
**Time from cue*Age**	4,124	1,445	0.223	0.045
**Time from cue*Group**	4,124	0.714	0.584	0.023
**Condition*Time from cue**	4,124	0.279	0.891	0.009
**Condition*Time from cue*Age**	4,124	0.053	0.995	0.002
**Condition*Time from cue*Group**	4,124	1,815	0.130	0.055
**Age**	1,31	1.969	0.170	0.060
**Group**	1,31	5.567	0.025	0.152

Supplementary table 9: Results of post-hoc t-tests between corticospinal excitability at time-points after the cue between critical and non-critical go trials

Time (ms)	t	p	d
**200**	-2.62	0.013	0.42
**250**	-4.24	< 0.001	0.61
**300**	-4.32	< 0.001	0.56
**350**	-2.37	0.024	0.20
**400**	-0.27	0.789	0.04

Supplementary table 10: Results of the repeated measures ANCOVA, adjusted for age, assessing the evolution of response-locked corticospinal excitability

Factor	dF	F	p	η^2
**Condition**	1,18	0.346	0.564	0.019
**Condition*Age**	1,18	0.447	0.512	0.024
**Condition*Group**	1,18	2.696	0.118	0.130
**Time from response**	4,72	8.998	< 0.001	0.333
**Time from response*Age**	4,72	1.491	0.214	0.077
**Time from response*Group**	4,72	0.863	0.491	0.046
**Condition*Time from response**	4,72	0.808	0.524	0.043
**Condition*Time from response*Age**	4,72	1.193	0.321	0.062
**Condition*Time from response*Group**	4,72	2.284	0.069	0.113
**Age**	1,18	2.475	0.133	0.121
**Group**	1,18	0.001	0.974	< 0.001

Supplementary table 11: Post-hoc unpaired (TD vs HC) t-tests between corticospinal excitability measured in the response-locked analysis

	Critical			Non-critical		
**Time before response (ms)**	**t**	**p**	**d**	**t**	**p**	**d**
**0-50 ms**	-1.78	0.091	0.65	-1.58	0.130	0.58
**50-100 ms**	-1.16	0.257	0.40	-0.49	0.631	0.16

Supplementary table 12: Spearman's rank correlation between YGTSS scores and critical/non-critical response-locked MEP amplitudes

Condition	Critical				Non-critical			
Time prior response (ms)	0-50		50-100		0-50		50-100	
YGTSS feature	r_s	p	r_s	p	r_s	p	r_s	p
**Motor**	-0.07	0.799	-0.25	0.328	-0.38	0.152	-0.21	0.409
**Vocal**	-0.05	0.864	0.31	0.227	0.06	0.828	0.27	0.28
**Severity**	< -0.01	0.990	0.07	0.793	-0.02	0.931	0.09	0.713
**Impairment**	< -0.01	0.995	0.05	0.858	0.19	0.490	0.07	0.786
**Total**	0.02	0.955	0.04	0.885	0.01	0.978	0.05	0.836

Supplementary table 13: Results of the repeated measures ANOVA assessing effects of SOA and Compatibility during the masked-priming task for TD and healthy control subjects

**Factor**	**dF**	**F**	**p**	**η^2**
**SOA**	7,294	126.8	< 0.001	0.751
**SOA*Group**	7,294	1.835	0.08	0.042
**Compatibility**	1,42	10.66	0.002	0.202
**Compatibility*Group**	1,42	5.93	0.019	0.124
**SOA*Compatibility**	7,294	4.31	< 0.001	0.093
**SOA*Compatibility*Group**	7,294	1.714	0.105	0.039
**Group**	1,42	0.708	0.405	0.017

Supplementary table 14: Results of the repeated measures ANOVA assessing effects of SOA and Compatibility during the masked-priming task for healthy control subjects

**Factor**	**dF**	**F**	**p**	**η^2**
**SOA**	7,175	80.52	< 0.001	0.76
**Compatibility**	1,25	0.36	0.553	0.01
**SOA*Compatibility**	7,175	3.02	0.005	0.11

Supplementary table 15: Results of the repeated measures ANOVA assessing effects of SOA and Compatibility during the masked-priming task for TD patients

Factor	dF	F	p	η^2
**SOA**	7,119	52.28	< 0.001	0.76
**Compatibility**	1,17	18.06	0.001	0.52
**SOA*Compatibility**	7,119	2.72	0.012	0.14

Supplementary table 16: Paired t-tests between compatible and incompatible trials for each SOA, for TD patients and healthy controls

	Primary tic disorder patients			Healthy controls		
**SOA (ms)**	**t**	**p**	**d**	**t**	**p**	**d**
**0**	-3.69	0.002	-0.40	0.494	0.625	0.06
**16**	-3.60	0.002	-0.37	-0.928	0.362	-0.01
**32**	-1.93	0.071	-0.21	0.627	0.536	0.04
**48**	-2.60	0.019	-0.32	0.173	0.864	0.01
**100**	-0.66	0.515	-0.06	2.457	0.021	0.10
**150**	-0.14	0.892	-0.02	-0.926	0.363	-0.07
**200**	-3.20	0.005	-0.39	-1.288	0.210	-0.11
**250**	-4.27	0.001	-0.45	-2.081	0.048	-0.22

Supplementary table 17: One-way ANOVA comparing compatibility effects between TD patients and healthy controls

SOA (ms)	dF	F	p
**0**	1,43	9.951	0.003
**16**	1,43	3.085	0.086
**32**	1,43	4.246	0.046
**48**	1,43	4.909	0.032
**100**	1,43	3.484	0.069
**150**	1,43	0.213	0.647
**200**	1,43	3.289	0.077
**250**	1,43	0.972	0.330

Supplementary table 18: Spearman rank correlations between masked prime task error types and clinical YGTSS scores. Severity = motor + vocal, Total = impairment + severity

	Motor		Vocal		Severity		Impairment		Total	
**Error type**	r_s	p	r_s	p	r_s	p	r_s	p	r_s	p
**Total**	0.53	0.024	0.42	0.085	0.50	0.036	-0.14	0.575	0.27	0.289
**Discrimination**	0.53	0.024	0.42	0.085	0.52	0.026	-0.15	0.562	0.26	0.292
**Fast**	0.58	0.012	0.35	0.161	0.48	0.046	-0.15	0.552	0.22	0.389
**Premature**	0.50	0.034	0.54	0.020	0.56	0.017	-0.15	0.549	0.316	0.202
**Impulsive (fast+premature)**	0.61	0.008	0.50	0.036	0.57	0.014	-0.14	0.570	0.31	0.211
**Omission**	0.33	0.180	0.10	0.700	0.11	0.673	-0.11	0.666	0.05	0.857

Supplementary table 19: Unpaired t-tests for YGTSS scores between patients with and without OCD/ADHD/depression/anxiety, and mood-enhancing medication

	OCD		ADHD		Depression		Anxiety		Medication use	
**YGTSS feature**	**t**	**p**	**t**	**p**	**t**	**p**	**t**	**p**	**t**	**p**
**Motor**	1.60	0.131	-2.02	0.067	-0.99	0.337	0.45	0.659	1.37	0.210
**Vocal**	0.33	0.753	-1.90	0.079	-0.56	0.587	0.11	0.912	1.21	0.249
**Severity**	0.84	0.418	-2.22	0.044	-0.80	0.438	0.27	0.791	1.36	0.208
**Impairment**	0.29	0.780	-0.41	0.692	-0.37	0.714	0.53	0.183	-1.60	0.145
**Total**	0.66	0.533	-1.85	0.091	-0.82	0.422	0.45	0.442	-0.25	0.806

Supplementary table 20: Unpaired t-tests for error types in the masked priming task between patients with and without OCD/ADHD/depression/anxiety, and mood-enhancing medication

	OCD		ADHD		Depression		Anxiety		Medication	
**Error type**	**t**	**p**	**t**	**p**	**t**	**p**	**t**	**p**	**t**	**p**
**Total**	0.36	0.724	1.37	0.210	-1.58	0.330	-0.03	0.979	0.48	0.643
**Discrimination**	0.58	0.575	1.21	0.249	-1.90	0.256	-0.04	0.969	0.86	0.415
**Fast**	0.15	0.882	1.36	0.208	-0.84	0.547	-0.11	0.915	-0.39	0.704
**Premature**	0.08	0.939	-1.60	0.145	-0.74	0.587	-0.23	0.818	-0.88	0.395
**Impulsive (fast+premature)**	0.13	0.903	-0.25	0.806	-0.80	0.562	-0.15	0.880	-0.56	0.587
**Omission**	0.12	0.911	-1.00	0.345	-1.15	0.453	-0.90	0.383	1.67	0.153

Supplementary table 21: Unpaired t-tests for priming effects (PCE/NCE) in the masked priming task between patients with and without OCD/ADHD/mood-enhancing medication

	OCD		ADHD		Depression		Anxiety		Medication	
**SOA (ms)**	**t**	**p**	**t**	**p**	**t**	**p**	**t**	**p**	**t**	**p**
**0**	-1.20	0.255	1.14	0.279	0.10	0.939	-1.42	0.205	-1.63	0.137
**16**	-1.21	0.243	1.89	0.079	0.11	0.931	-0.28	0.785	-0.89	0.394
**32**	-0.51	0.616	2.32	0.034	-0.46	0.722	-0.42	0.692	-1.22	0.247
**48**	-0.66	0.520	0.46	0.66	1.02	0.483	0.63	0.542	-1.02	0.340
**100**	0.61	0.557	0.70	0.494	0.14	0.896	1.07	0.324	-1.04	0.315
**150**	0.40	0.694	-1.10	0.301	-0.72	0.599	0.59	0.566	1.04	0.336
**200**	0.24	0.82	-0.16	0.875	0.25	0.841	1.43	0.198	0.64	0.537
**250**	-1.76	0.098	-0.71	0.490	-3.42	0.096	0.05	0.963	1.15	0.286
